# The influence of regional preferential trade agreements on international manufacturing trade in value-added: Based on the complex network method

**DOI:** 10.1371/journal.pone.0246250

**Published:** 2021-02-19

**Authors:** Bo Wang, Yue Pu, Shunli Li, Lin Xu

**Affiliations:** International Business School, Southwestern University of Finance and Economics, Chengdu, China; Scuola Superiore Sant’Anna, ITALY

## Abstract

Based on a new trade accounting method—the trade in value-added accounting method—this paper constructs the international manufacturing trade in value-added networks and preferential trade agreement (PTA) networks and uses the complex network analysis method to explore the relations between PTA and international manufacturing trade in value-added from the perspective of the global value chain. The results are as follows: (1) Over the years, the international manufacturing trade in value-added networks and PTA networks has shown a significant clustering effect, and the size of networks has grown rapidly. (2) The TEX, DVA and FVA networks of the international manufacturing value added trade over the years can be divided into two societies in the Asia-Pacific region and the European region. This division just reflects the different modes of division of labor in the manufacturing value chain of the two major economic regions in the world. (3) QAP analysis shows that the influencing factors of the traditional gravity model can still explain the manufacturing trade network and its value-added trade network, while the influence of economic globalization, the enlargement of the EU and the internationalization strategy of enterprises, the PTA network and manufacturing value-added the relationship between trade networks changed from positive to negative in 2004.

## Introduction

In the context of the profound adjustment of the global economy, the frequent trend of "deglobalization" and the stagnation of multilateral trade negotiations, the rules of regional investment and trade have changed, and a new round of trade and investment agreement negotiations have been launched around the world, such as the TPP, TTIP, TISA and a large number of bilateral trade agreements. According to the data in December 2017, 249 preferential trade agreements (PTAs) had been notified to the World Trade Organization (WTO) and come into force. With the emergence of increasing numbers of multilateral and regional preferential trade agreements (PTAs), researchers have conducted abundant research on trade agreements [1–15].

Among them, whether trade agreements have a significant impact on the flow and direction of international trade has been a hot topic in the field of international trade. Previous research was mainly based on traditional gross trade statistics and used the traditional gravity model [16–25], matching [26], computable partial equilibrium model [27], Matching and panel fixed effects [28] and meta-analysis [29] to explore the relation between trade agreements and international trade. However, with the improvement of specialization in the global value chain and the increasingly frequent trade in intermediate goods, traditional statistics on total trade volume can no longer truly reflect the trade gains of a country and tend to exaggerate the value created by a country in its export [30].

Therefore, some researchers reanalyzed the international trade pattern based on the method of measuring trade in value-added from the perspective of the global value chain. Hummel et al. [31] defined vertical specialization and proposed the HIY value chain measuring method. Daudin et al. [32] further proposed the DRS method on the basis of the HIY method, which can measure the share of domestic value-added after processing in the direct importing country and sale back to the home country. On the basis of the HIY and DRS methods, Koopman et al. [33] relaxed the key assumptions of the HIY method and initially proposed the theory of measuring trade in value-added. On this basis, Wang et al. [30] further improved the method of measuring trade in value-added at the bilateral and sector levels, forming the theoretical foundation of measuring trade in value-added.

Over the years, PTAs and international trade have each formed a complex global network. In this case, the traditional trade indicators cannot comprehensively and systematically reflect the complex trade relations between countries [34]. Through the detailed description of the network topology, the complex network analysis method makes up for the deficiency of the traditional trade indicators and can analyze the features of international trade networks scientifically and comprehensively [35]. For example, Sopranzetti & Silvia [36] used the network method to study the impact of a country’s position in the FTA network on bilateral trade and found that the hub-and-spoke characteristics of FTA have a positive and significant impact on the bilateral trade of the central country, but the study still used bilateral trade data through the traditional trade measuring method. Although Zhou et al. [37] explored China’s previous trade relations and sector status relative to EU member states based on trade in value-added and complex network analysis methods, the study only built international trade networks and did not build trade agreements networks from the perspective of network relations or quantitatively analyze the impact of trade agreements on trade networks.

To fill the gap in the current literature, our paper aims to apply WWZ trade in the value-added accounting method and the complex network method to analyze the network topology structural features and evolutionary patterns of global PTA networks and manufacturing export networks over the period of 2000–2014 from the perspective of the global value chain and use the QAP model to explore the impact of PTAs on the global manufacturing industry from the network relation level. Overall, our paper contributes to the literature in the following three ways. First, from the perspective of the global value chain, our paper applies the new accounting system of value-added measurement to redraw the pattern of international manufacturing trade. Second, based on the complex network analysis method, our paper investigates the structural features of PTA networks and international manufacturing trade networks. Third, our paper uses the QAP network quantitative method, which is specifically aimed at relational data, to analyze the impact of PTA on international manufacturing trade.

The remainder of the paper is organized as follows: Section 2 describes the FTA and manufacturing trade data and introduces the WWZ accounting method, network feature indicators and the QAP model. Section 3 presents the results of the network feature measurement and the analysis of influencing factors. Section 4 summarizes the results.

## Data and methodology

### Data

In this paper, the PTA data are from the preferential trade agreements database of the World Bank [38]. The database covers 279 agreements signed by 189 countries between 1958 and 2015, which reflects the entire set of preferential trade agreements in force and notified to the World Trade Organization in 2015. The data on manufacturing trade are from the most recent world input-output table published by the World Input-Output Database (WIOD), covering 56 sectors in 43 important economies (S1 Table) from 2000 to 2014. Sectors 5–22 in the world input-output table are selected as manufacturing industries, as shown in Table 1.

**Table 1:** 18 Manufacturing sectors in this paper.

No.	Manufacture	No.	Manufacture
5	Manufacture of food products, beverages and tobacco products	14	Manufacture of other non-metallic mineral products
6	Manufacture of textiles, wearing apparel and leather products	15	Manufacture of basic metals
7	Manufacture of wood and of products of wood and cork, except furniture; manufacture of articles of straw and plaiting materials	16	Manufacture of fabricated metal products, except machinery and equipment
8	Manufacture of paper and paper products	17	Manufacture of computer, electronic and optical products
9	Printing and reproduction of recorded media	18	Manufacture of electrical equipment
10	Manufacture of coke and refined petroleum products	19	Manufacture of machinery and equipment n.e.c.
11	Manufacture of chemicals and chemical products	20	Manufacture of motor vehicles, trailers and semi-trailers
12	Manufacture of basic pharmaceutical products and pharmaceutical preparations	21	Manufacture of other transport equipment
13	Manufacture of rubber and plastic products	22	Manufacture of furniture; other manufacturing

Note: The numbers in the table are the numbers of sectors in the WIOD.

### Methodology

#### The decomposition of value-added manufacturing exports

With the improvement in the specialization in the global value chain, traditional statistics on total trade volume can no longer truly reflect the trade gains of a country, and the rapid growth of international trade in intermediate goods tends to exaggerate the value created by a country in its export [32]. Therefore, we use the WWZ accounting system proposed by Wang et al. [30], which extends the method of decomposing a country’s gross trade flows put forward by Koopman et al. [33], dividing intermediate trade flows at all levels (economy/sectoral levels, bilateral levels, bilateral sectoral levels) into sixteen pathways depending on tradable goods’ value source, territory of final absorption and absorption channels. In doing so, the decomposition of bilateral intermediate trade flows is completely achieved. Taking the trade of three countries as an example, Table 2 shows the input-output model among three countries (S, R, T).

**Table 2:** The input-output model of three countries.

output input		intermediate use			final use			total output
		S	R	T	S	R	T	
intermediate input	S	Z^ss^	Z^sr^	Z^st^	Y^ss^	Y^sr^	Y^st^	X^s^
	R	Z^rs^	Z^rr^	Z^rt^	Y^rs^	Y^rr^	Y^rt^	X^r^
	T	Z^ts^	Z^tr^	Z^tt^	Y^ts^	Y^tr^	Y^tt^	X^t^
value-added		VA^s^	VA^r^	VA^t^	—	—	—	—
total input		(X^s^)’	(X^r^)’	(X^t^)’	—	—	—	—

Superscripts s, r, and t in the table represent country s, country r, and country t, respectively. Let Z^sr^ and Y^sr^ represent the intermediate input and final use of country R, respectively, and VA^s^ and X^s^ represent the value-added and output of country S, and so on. The superscript “’” is transpose. Assuming that the number of national sectors is unified into n, then in the above table, Z is a matrix of n*n, X and Y are the column vectors of n*1, and V is the row vector of 1*n. According to the WWZ accounting method, after completely decomposing the bilateral intermediate trade flows and introducing the value-added coefficient, the E^sr^, which represents the exports from country S to country R, can be divided into the following 16 value-added and double-counted parts depending on their value source and territory of final absorption:

$$\begin{array}{l} E^{sr}=\underset{DVA}{\underset{︸}{\begin{array}{l} \left(V^{S}B^{SS}\right)'\#Y^{Sr}+\left(V^{S}L^{SS}\right)'\#\left(A^{Sr}B^{rr}Y^{rr}\right)+\left(V^{S}L^{SS}\right)'\#\left(A^{Sr}B^{rt}Y^{tt}\right)\\ +\left(V^{s}L^{ss}\right)'\#\left(A^{sr}B^{rr}Y^{rt}\right)+\left(V^{s}L^{ss}\right)'\#\left(A^{sr}B^{rt}Y^{rt}\right) \end{array}}}\\ \;\underset{FVA}{\underset{︸}{+\left(V^{r}B^{rs}\right)'\#Y^{sr}+\left(V^{r}B^{rs}\right)'\#\left(A^{sr}L^{rr}Y^{rr}\right)+\left(V^{t}B^{ts}\right)'\#Y^{sr}+\left(V^{t}B^{ts}\right)'\#\left(A^{sr}L^{rr}Y^{rr}\right)}}\\ \;\underset{RDV}{\underset{︸}{+\left(V^{s}L^{ss}\right)'\#\left(A^{sr}B^{rr}Y^{rs}\right)+\left(V^{s}L^{ss}\right)'\#\left(A^{sr}B^{rt}Y^{ts}\right)+\left(V^{s}L^{ss}\right)'\#\left(A^{sr}B^{rs}Y^{ss}\right)}}\\ \;\underset{PDC}{\underset{︸}{\begin{array}{l} +(V^{s}L^{ss})'\#[\left(A^{sr}B^{rs\left(Y^{sr}+Y^{st}\right)}\right]+\left(V^{s}B^{ss}-V^{s}L^{ss}\right)'\#\left(A^{sr}X^{r}\right)\\ +\left(V^{r}B^{rs}\right)'\#\left(A^{sr}L^{rr}E^{r}\right)+\left(V^{t}B^{ts}\right)'\#\left(A^{sr}L^{rr}E^{r}\right) \end{array}}} \end{array}$$ (1)

**Fig 1:**
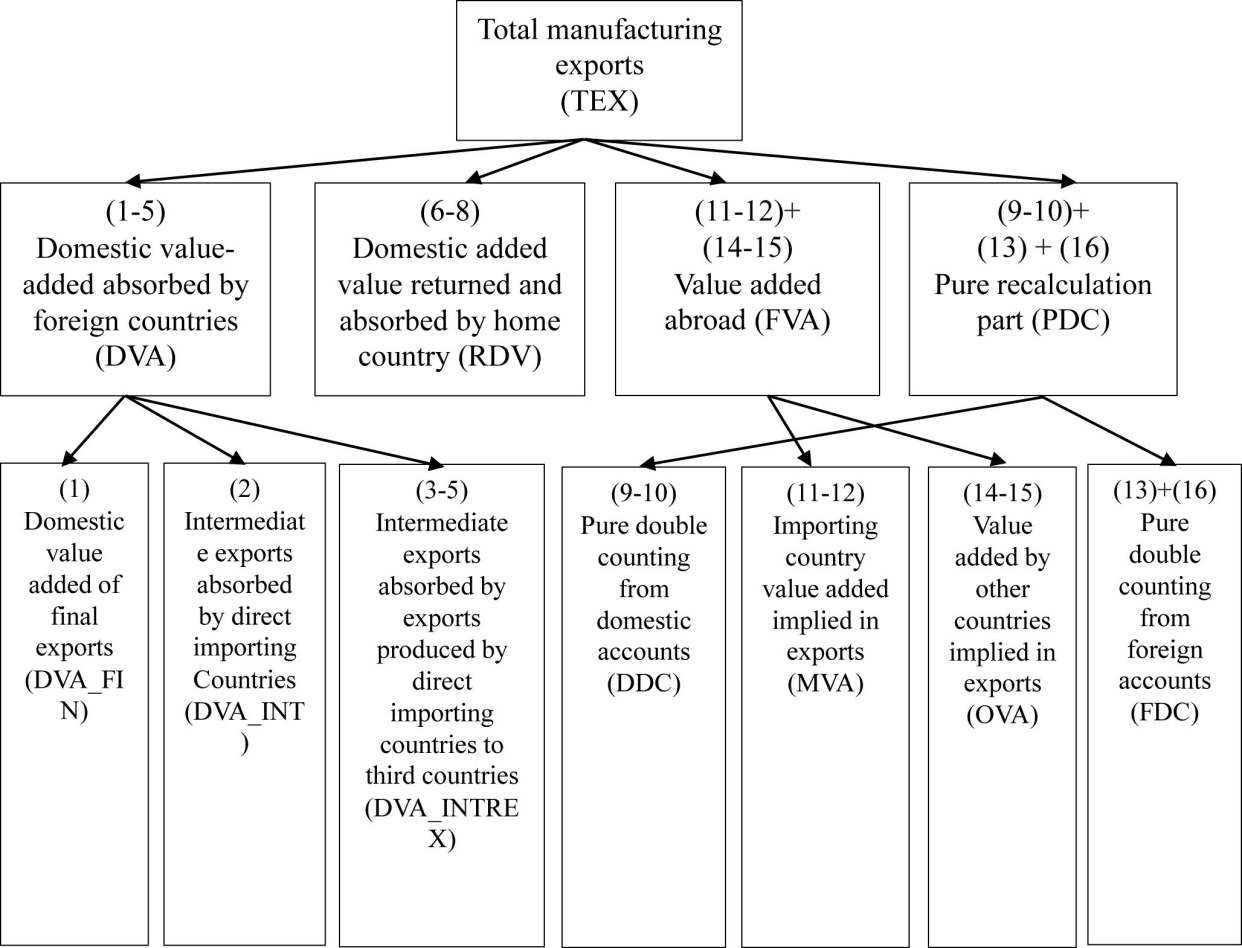
Decomposition framework of WWZ trade value added of manufacturing total exports based on Wang (2014).

In summary, the relationship between the 16 decomposition parts of manufacturing trade exports can be seen in Fig 1. The subdivision data of bilateral manufacting trade in 2000 and 2014 are shown in S1 and S2 Data, respectively.

**Fig 2:**
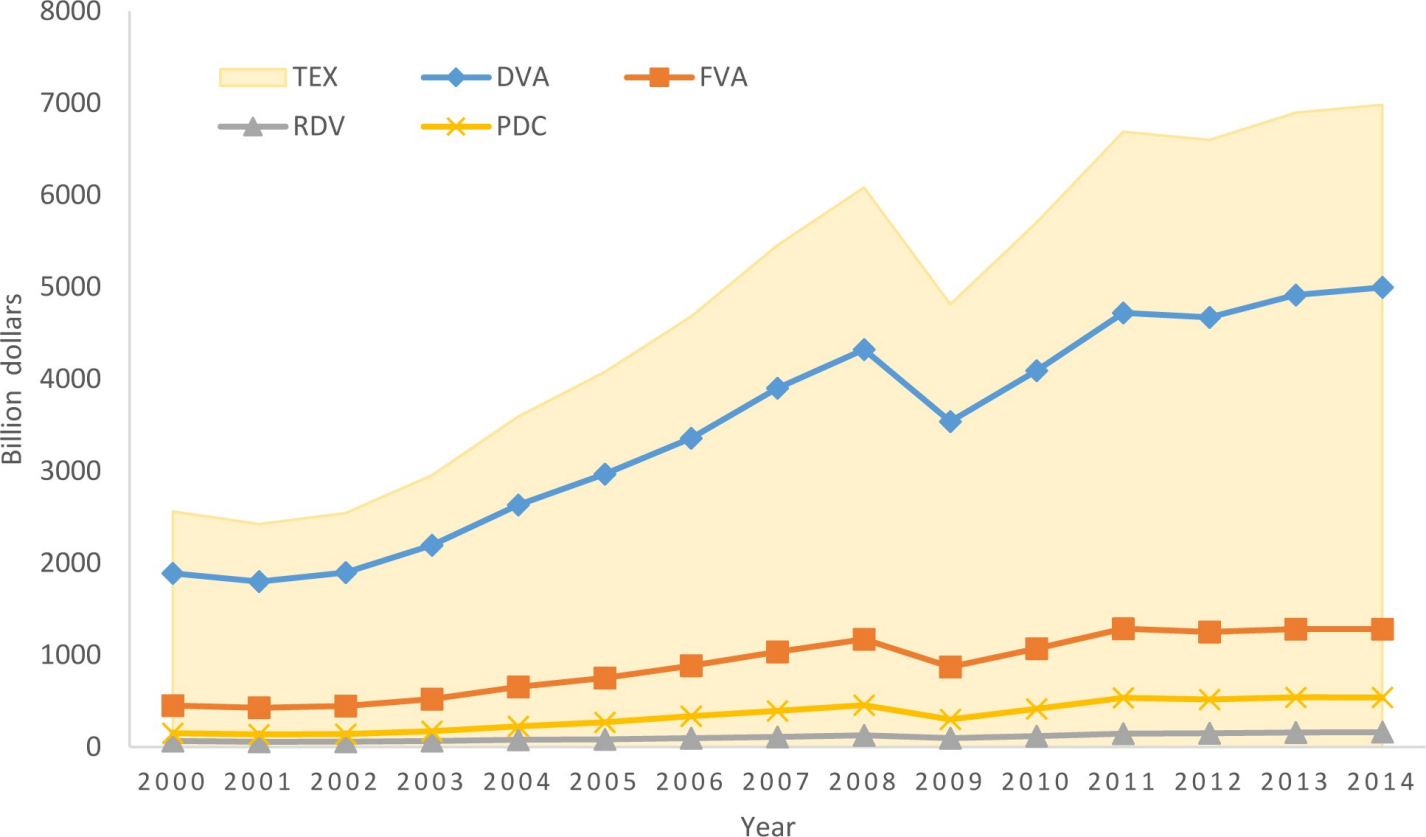
The decomposition results of international manufacturing trade in value-added over the years.

Based on the above method, we decompose international manufacturing trade flows into domestic value-added (DVA), foreign value-added (FVA), returning domestic value-added (RDV) and purely double-counted trade in intermediate goods (PDC). Fig 2 shows the decomposition results of international manufacturing trade in value-added over the years. Because the sum of value-added in domestic and foreign exports accounting for manufacturing trade as high as 90%, domestic value-added is the direct source of the profits of a country’s participation in international trade, and foreign value-added is an important symbol of integration into the global value chain and participation in international specialization, we focus on DVA and FVA to analyze the pattern of manufacturing trade in value-added and its evolution.

**Fig 3:**
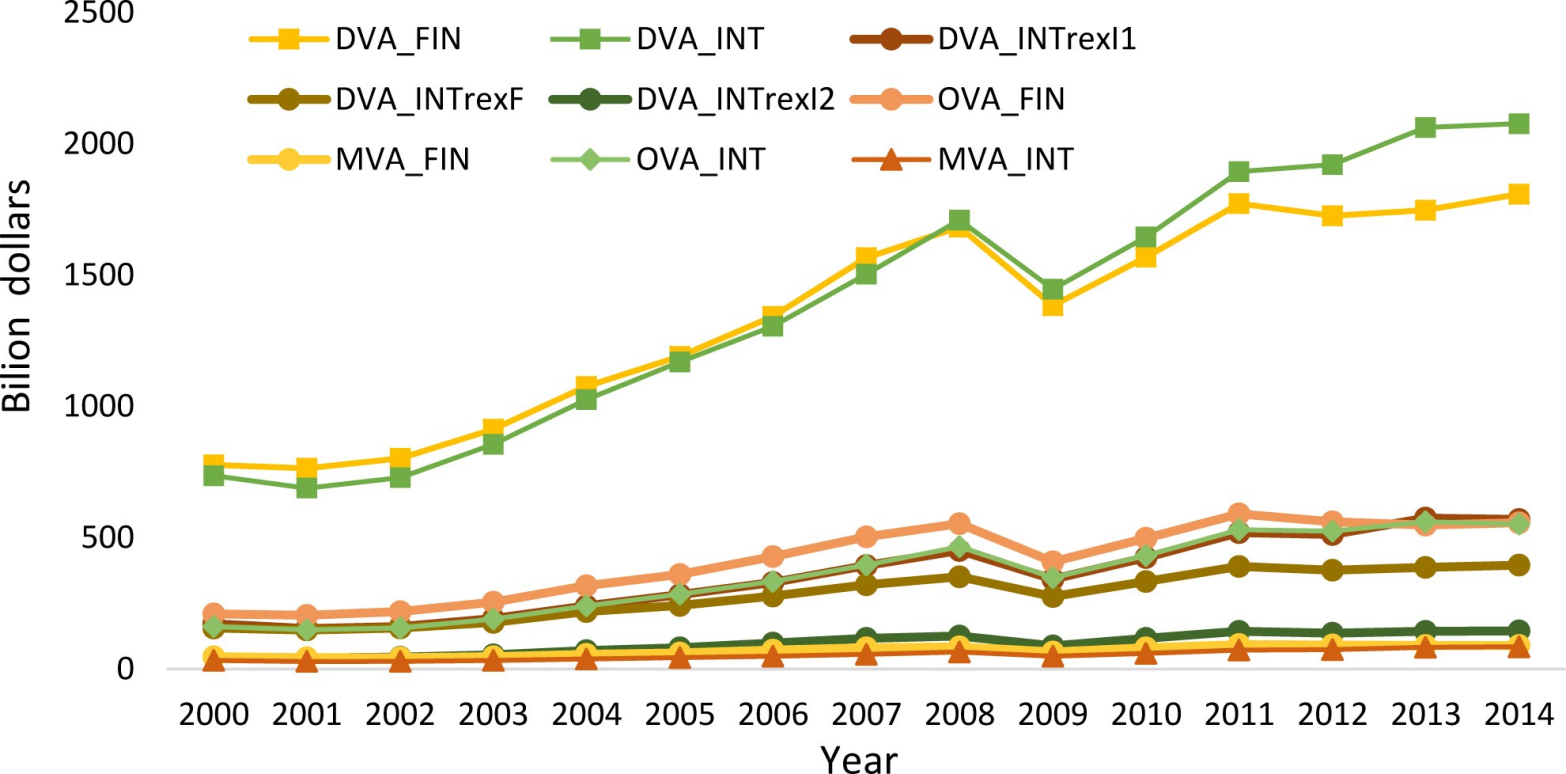
The subdivisions of DVA and FVA in manufacturing trade added value from 2000 to 2014.

Fig 3 shows the nine subdivisions of DVA and FVA in the manufacturing trade value added from 2000 to 2014. Combining Figs 1 and 3, we can see that the final domestic value added of exports in DVA and the intermediate absorbed by direct importing countries Exports account for a large proportion; In FVA, the value-added of importing countries is mainly domestic exports, and the third-country added value of domestic exports accounts for a small proportion. This shows that in manufacturing trade, intermediate goods trade mainly occurs between direct exporting countries and direct importing countries, and there are relatively few intermediate goods trades involving third countries, that is, in the manufacturing industry value chain, bilateral cooperation is more common, The three or even multi-country cooperation is rare. The three or multi-country cooperation is mainly concentrated in a few powerful large-scale cross-manufacturing enterprises.

#### The construction of manufacturing trade in value-added networks

A complex network is a collection of nodes and their relations (edges). According to the complex network method, we take each economy as a node and the trade relations between economies as the edges. Based on the WWZ accounting method, trade flows are divided into four types of value-added flows. We mainly build and analyze the DVA networks and FVA networks but also give the total manufacturing exports (TEX) networks as a reference. Because the manufacturing trade flows between economies differ in trade direction and trade volume, our international manufacturing trade networks in this paper are weighted and directed. To better understand the features of network structure and node attributes, taking 10 million US dollars as the threshold value, we remove the edges below the threshold value in the complete network. It is concluded that the ratio of the threshold value network weight to the total network weight in each year is higher than 95%, which is fairly representative. The international manufacturing trade networks are shown in Fig 5. The subdivisions of DVA and FVA in 2014 are shown in S1 and S2 Figs, respectively.

#### The construction of PTA networks

The PTA networks in this paper take the economies that have signed preferential trade agreements as the nodes, the preferential trade agreements between two economies as the edge and the depth of preferential trade agreements between economies as the edge weight. Since the preferential trade agreement relations are essentially reciprocal trade arrangements between economies that are undirected, our PTA networks in this paper are undirected and weighted. To measure the depth of PTA rules, referring to the study of international law experts of the World Bank, Hofmann et al. [38], we divide PTA rules into WTO + clauses (first-generation trade rule clauses) and WTO-X clauses (second-generation trade rule clauses) and 52 subcategories and then construct the quantitative index of PTA rule depth. In terms of the quantitative calculation of PTA rule depth, we construct the index of rule depth (*IDep*) by referring to the rule quantization calculation formula proposed by Kohl et al. (2016) [40], as shown in formula 2.

$$IDep_{m}=\sum_{i=1}^{52}\omega_{i}$$ (2)

**Fig 4:**
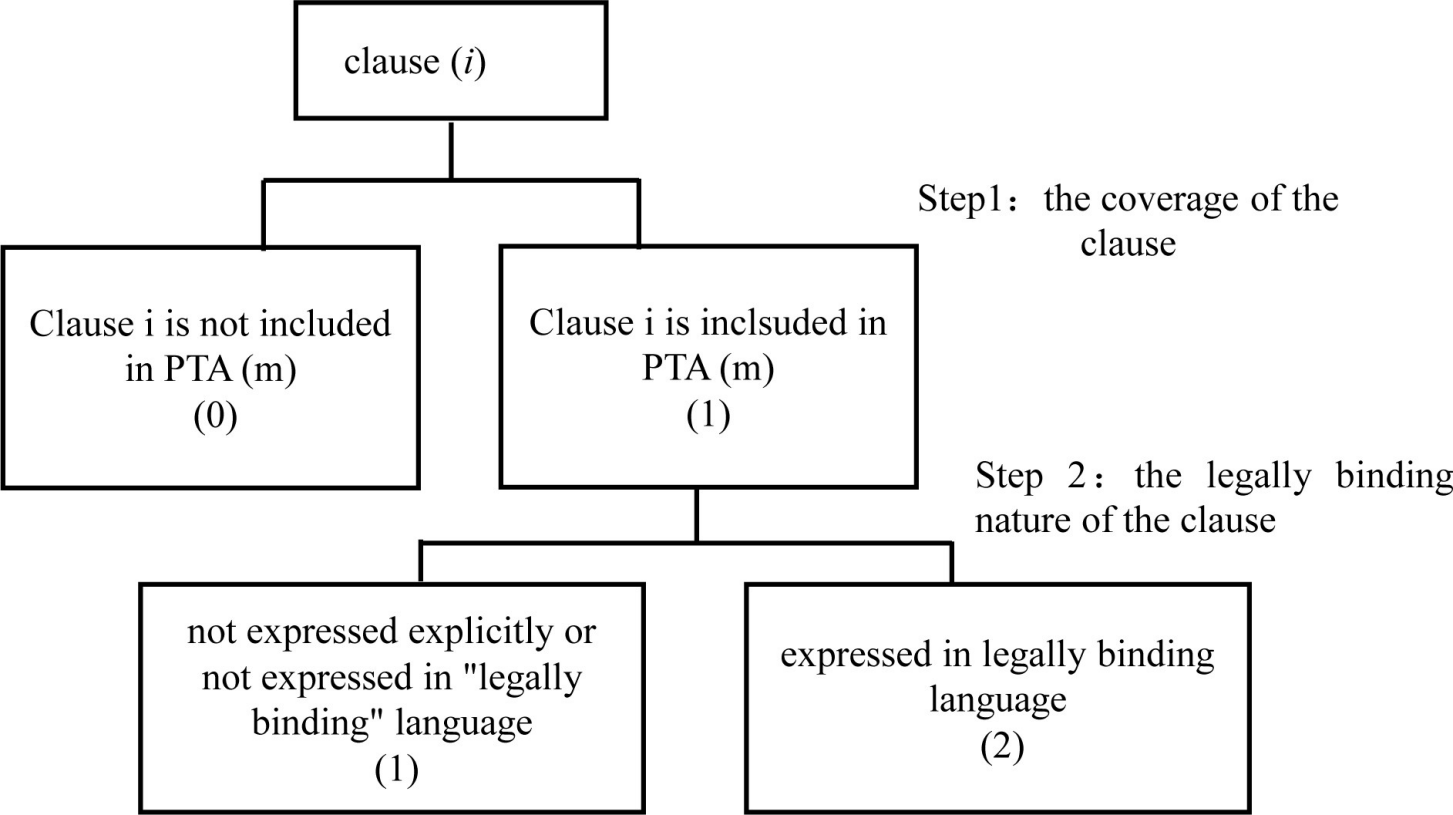
Quantitative scoring method of the depth of PTA.

**Fig 5:**
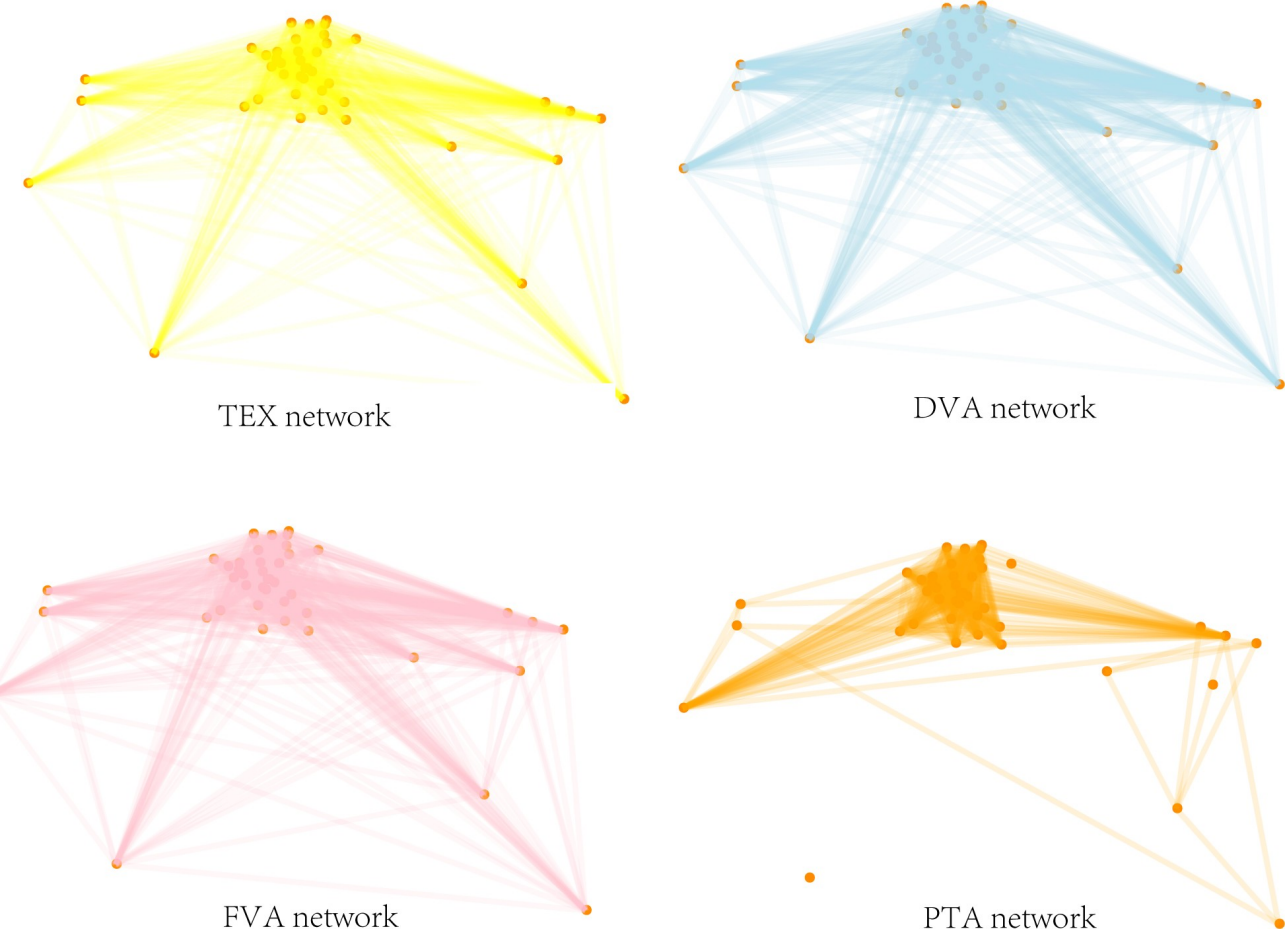
International manufacturing trade networks and PTA network in 2014.

*m* represents a certain PTA, and *i* represents the 52 subcategories of trade agreement provisions and rules mentioned above, *i* = 1, 2, 3, …, 52. *ω*_*i*_ represents the depth of rules corresponding to class i clauses in a PTA. The scoring method refers to the study of international law experts Hofmann et al. [38] of the World Bank; that is, we first consider whether the PTA covers category i clauses or not; if not, it scores “0”; it scores “1” if it covers category i clauses but is not expressed explicitly or in "legally binding" language; it scores “2” if it covers category i clauses and is expressed in legally binding language (Fig 4). Then, the corresponding scores of 52 categories in the PTA are added up, and the sum is the weight of the international preferential trade agreement (PTA) network. The PTA networks are shown in Fig 5.

### Methodology

#### (1) Network feature measurements

We use the following terms to describe the different features of international manufacturing trade in value-added networks and PTA networks.

*Numbers of network nodes and edges*. The numbers of nodes and edges of the network are the two primary network indicators for measuring the network size. The node number refers to the total nodes contained in the network. The number of edges is the quantity of all relations. In this paper, node number represents the number of economies in the networks, while edge number represents the number of relations of global manufacturing trade in value-added or signed PTAs.

*Network density*. It is the ratio of the edge number in the network to the number of all edges that have the same number of nodes, which can be used to measure the degree of closeness between network nodes and ranges from 0 to 1. When the density of a network is close to 1, it means that the network is dense, indicating that the network is closely related; instead, the closer the density is to 0, the sparser the network is, and the more alienated the network relations are from each other.

*The average path length*. The average path length of a network is the average distance between nodes, and it is often used to measure the effectiveness of information transfers between network nodes. In the networks, when the shortest distance between economy *i* and economy *j* is 1, it means these two economies are directly linked; if the shortest distance is 2, it means these two economies need to be connected through a third intermediate economy; the rest follow the same logic. Let *APL* represent the average path length of the network and *d*_*ij*_ represent the distance between node *i* and node *j* in the network. The formula of the average path length can be expressed as

$$APL=\frac{1}{N(N+1)}\sum_{i\geq j}d_{ij}$$ (3)

*The clustering coefficient*. The clustering coefficient is measured by the proportion of the triangle relations existing in the networks to all possible triangle relations, reflecting the degree to which the neighbors of node *i* are connected to each other.

$$\mathrm{Transitivity}=\frac{3\times M_{\mathrm{trangles}}}{M_{\mathrm{triples}}}$$ (4)

*M*_*triangles*_ represents the triangle relations existing in the networks, and *M*_*triples*_ represents all possible triangle relations in the networks.

*Node degree*. The node degree is the most important and direct indicator to show the importance of nodes. The node degree indicates the direct connection between a node and the rest of the nodes within the network, and a node with a high node degree is likely to act as a center for information exchange, thus demonstrating whether the node is at the core position of a network. The directed network of global manufacturing trade in value-added networks can be divided into in- and out-degrees according to the direction of the edge. The out-degree of a node is the directed edge number originating from it, and the in-degree of a node refers to the number of directed edges pointing to it. The formula is

$$k_{i}^{out}=\sum_{j=1}^{N}a_{ij},k_{i}^{in}=\sum_{j=1}^{N}a_{ji}$$ (5)

$k_{i}^{out}$. and $k_{i}^{in}$ are the out-degree and in-degree of a node, respectively; *a*_*ij*_ is a binary variable and is the element of the corresponding unweighted adjacent matrix of the network, where *a*_*ij*_ = 1 indicates that there is a directed edge from node i to node j; *a*_*ij*_ = 0 otherwise.

*Node strength*. Node strength demonstrates the closeness of the connection between nodes. In weighted directed networks of global manufacturing trade in value-added, the out-strength of a node is the sum of the weights of all directed edges pointing out from that node, and the in-strength is the sum of the weights of all directed edges pointing to the node. The formula is

$$s_{i}^{out}=\sum_{j=1}^{N}w_{ij},s_{i}^{in}=\sum_{j=1}^{N}w_{ji}$$ (6)

$s_{i}^{out}$ and $s_{i}^{in}$ are the out-strength and in-strength of a node, respectively, and *w*_*ij*_ is the weight of an edge between node *i* and node *j*, which is the export (import) volume from economy *i* to economy *j*.

*Normalized mutual information*. Normalized mutual information can be used to measure the similarity between two communities, and then it can be used to measure the degree of stability of communities in different years or the similarity of communities divided by different methods in the same year. By comparing the members in communities in different years, the stability of the communities in different years can be measured. The formula is:

$$\mathrm{NMI}(A,B)=\frac{‐2\sum_{h=1}^{k^{A}}\sum_{l=1}^{k^{B}}n_{h,l}\;\log\left(\frac{nn_{h,l}}{n_{h}^{A}n_{t}^{B}}\right)}{\sum_{h=1}^{k^{A}}n_{h}^{A}\;\log\frac{n_{h}^{A}}{n}+\sum_{l=1}^{k^{B}}n_{l}^{B}\;\log\frac{n_{l}^{B}}{n}}$$ (7)

A and B are the results of the two community division methods. *k*^*A*^ is the number of communities included in A. $n_{h}^{A}$ is the number of “economy-industry” of community h in A. *n*_*h*,*l*_ represents the number of “economy-industry” in both community h and mmunity l. n represents the number of all “economy-industry” in the networ.

#### (2) QAP regression model

The ordinary regression models require the assumption that samples and variables are independent of each other. As relational variables, network data itself reflects the relations between individuals, so these relational variables may be highly correlated with each other, which cannot satisfy the independence hypothesis of ordinary regression models. In this case, if the ordinary regression models are used for parameter estimation, the standard deviation of the parameter estimation will be too large, and the significance test of the variable will be meaningless. QAP is a nonparametric estimation method that is used to analyze the correlation between variables. It does not need to assume that independent variables are independent from each other, so the results are more robust than those of the parametric method.

There are three steps in the operation process of QAP. First, the independent variable matrix and dependent variable matrix are converted into a long vector, and the regression coefficient of the model is calculated by the OLS method. Second, the rows and columns of the dependent variable matrix are permutated randomly many times, OLS regression is conducted, and the coefficient and goodness of fit are saved each time. Finally, the saved coefficient and goodness of fit are ranked to form a reference distribution, and then the initial estimated statistics are compared with the values in the reference distribution to obtain the standard deviation of the estimated statistics. If less than 5% or 1% of the estimated coefficient values from the permutation regression are greater than the observed coefficient values, the coefficient can be judged to be significant at the level of 0.05 or 0.01 (one-tailed test).

## Results and discussion

### Overall structural feature analysis of the networks

**Fig 6:**
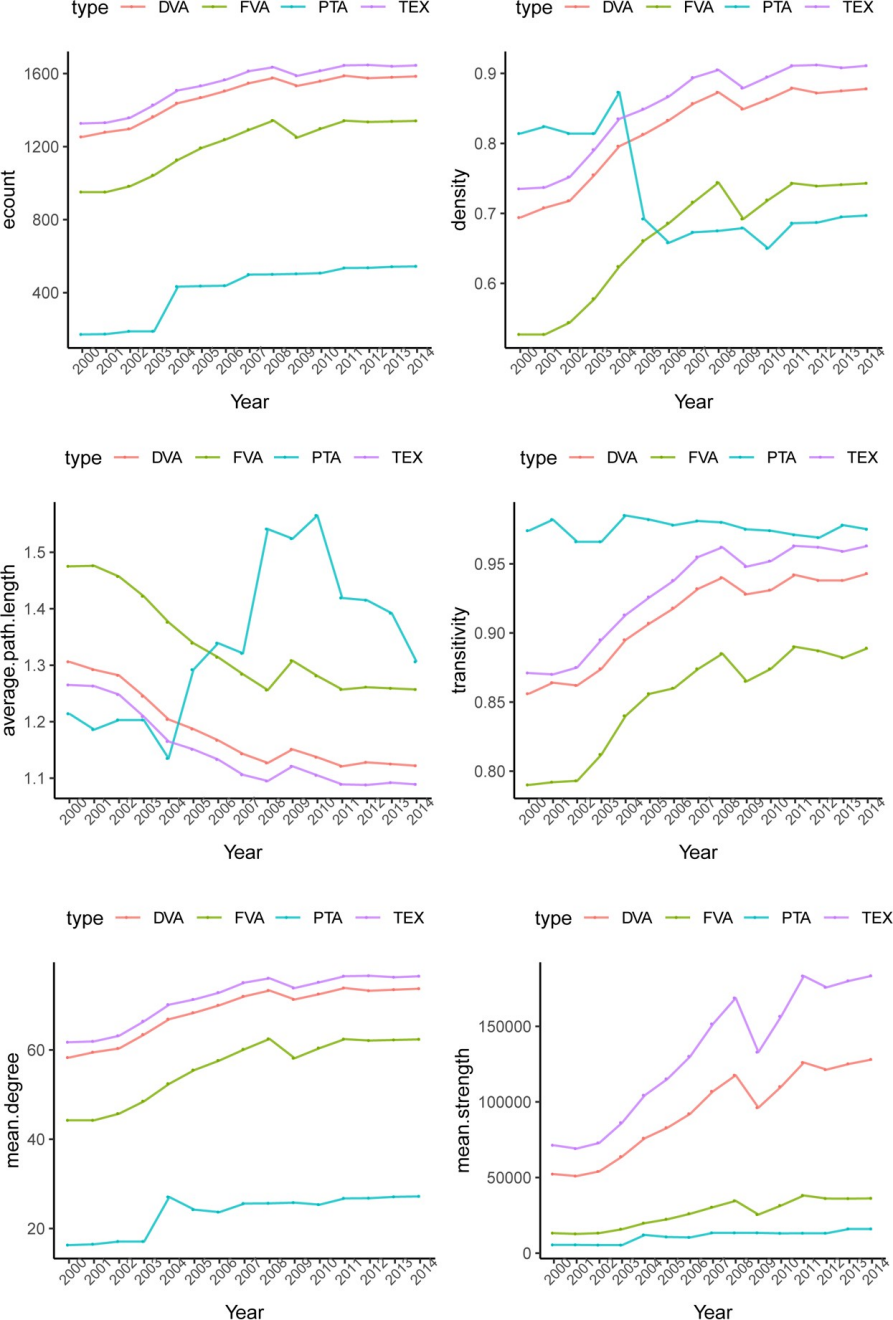
The overall features of international manufacturing trade in value-added networks and PTA networks. The unit of average strength is 10 million dollars.

Fig 6 shows some basic statistics for the international manufacturing trade in value-added networks and PTA networks from 2000 to 2014. From the perspective of network scale, the network scale of international manufacturing trade and PTA showed a trend of rapid growth over the years. In terms of the number of nodes, the node number of TEX networks, DVA networks and FVA networks of international manufacturing trade in value-added networks was 43, covering all the economies involved in the input-output data in this paper. The scale of PTA networks presented a growth trend, and the node number increased from 21 in 2000 to 40 in 2014, indicating the growing number of countries and regions signing preferential trade agreements. In terms of the number of edges in the networks, the edge number of both international manufacturing trade in value-added networks and PTA networks increased rapidly, especially the FVA networks and PTA networks. The number of edges in FVA networks increased from 951 in 2000 to 1341 in 2014, an increase of 41%, and the number of edges in PTA networks grew from 171 in 2000 to 544 in 2014, an increase of 218%. Furthermore, the average node degree and average node strength of the above four networks showed an overall growth trend, except for the impact of the expansion of EU members on the PTA network in 2004 and the impact of the 2008 financial crisis on the international manufacturing trade in the value-added network. This indicates that with the deepening of specialization in the world, economies are increasingly embedded in the global value chain. Moreover, reciprocal trade relations between economies are increasing.

From the perspective of the closeness of networks, the density and average path length of TEX, FVA and DVA networks of international manufacturing trade displayed a consistent trend of development. The density of the above networks accelerated and the average path length decreased rapidly around 2002, while the growth of density of the above networks slowed after the 2008 financial crisis, and the average path length decline also slowed. This shows that, influenced by China’s entry into the WTO and other events accelerating trade liberalization around 2002, with the rapid development of economic globalization, the manufacturing trade in value-added networks displayed a trend of accelerating denseness, and the accessibility and efficiency of trade relations accelerated. The financial crisis had a certain impact on the trade network of added value of the manufacturing industry. Under the influence of the financial crisis in 2008, the networks of manufacturing trade in value-added showed a sparse trend for a short time, and they gradually resumed dense development in the post-financial crisis period. The clustering coefficient is large, and the average clustering coefficients of the TEX networks, DVA networks and FVA networks of international manufacturing trade in value-added networks are 0.93, 0.91, 0.85 and 0.98 over the years, indicating that the above four networks have obvious clustering effects. Moreover, the clustering coefficient rose from 2000 to 2014, illustrating that the global manufacturing trade market and international preferential trade agreements are becoming increasingly collectivized, and the trend of regional cooperation is strengthening.

### The community analysis of the networks

After analyzing the overall pattern of the networks, in this part, we adopt the community analysis method of complex networks to depict the communities formed in the international manufacturing trade in value-added networks and PTA networks depending on the density of relations between economies. We use the walktrap method of complex networks to divide the communities in TEX networks, DVA networks, FVA networks of international manufacturing trade and PTA networks and then use the normalized mutual information index to analyze the stability of communities in different networks and the similarity of networks divided by different methods.

**Fig 7:**
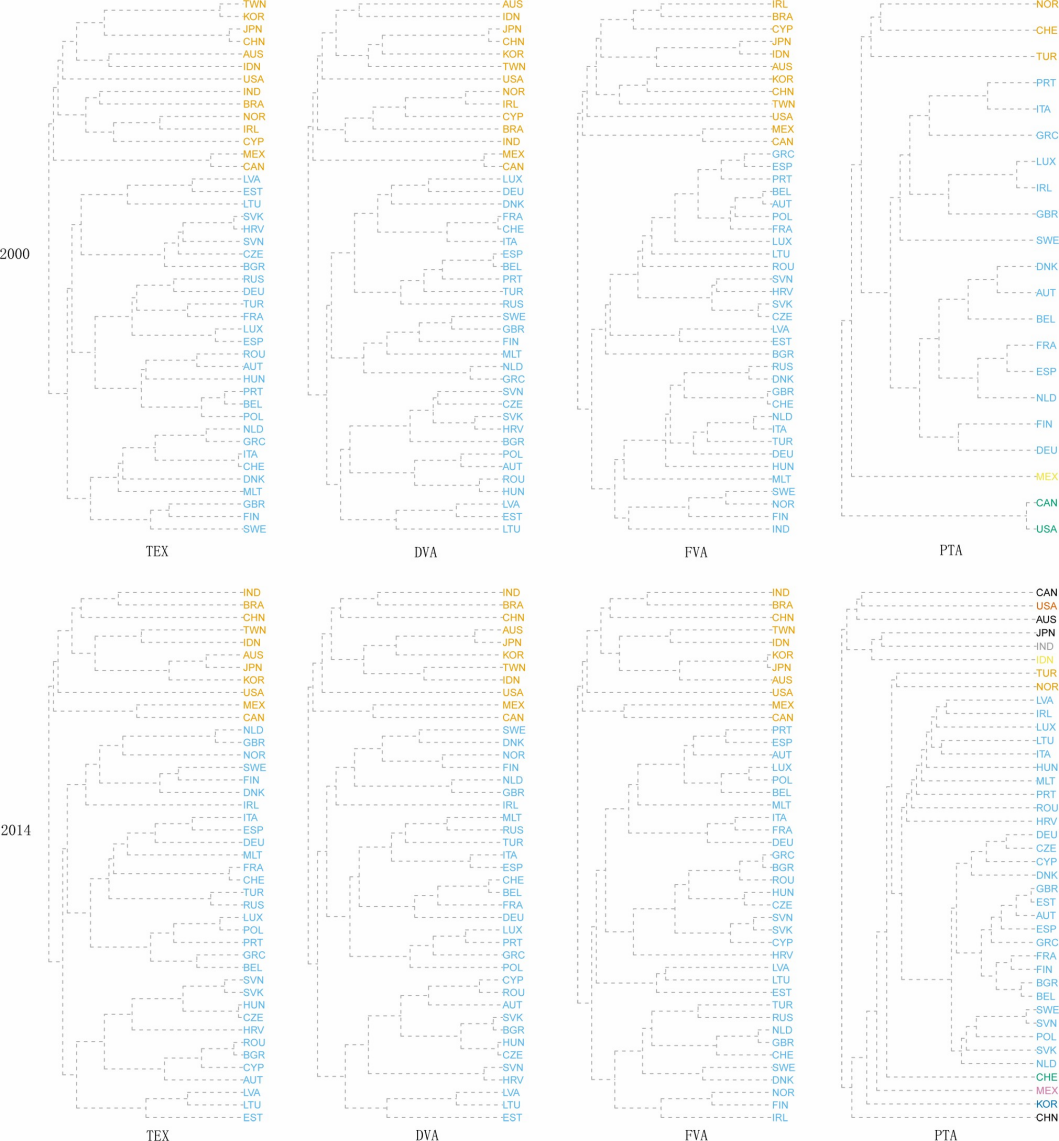
Tree view of the community division in 2000 and 2014.

As shown in Fig 7, over the years, the TEX networks, DVA networks and FVA networks of international manufacturing trade in value-added can be divided into two communities. In the early stage, there were certain differences in the members of the communities in the above three networks. In 2014, the TEX, DVA and FVA networks were divided into community 1, including India, Brazil, mainland China, Taiwan China, Indonesia, South Korea, Japan, Australia, the United States, Mexico, and Canada, a total of 11 economies, mainly emerging economies as well as North America and East Asia, and community 2, including 32 economies represented by Germany, France and other EU economies. The division of community 1 and community 2 exactly reflects the different division of labor in the manufacturing value chain of the two major economic regions in the world. The countries in Association 1 are mainly distributed in the Asia-Pacific region, and the factor endowments of each economy are obviously different. Through direct investment, cross-border production cooperation and intermediate trade of parts and components, the continuous refinement of the division of manufacturing has made countries bear different factor-intensive characteristics. In the production process, trade associations are becoming closer. It is clearly different from the community 1. Due to the geographical proximity and the convenience of language and currency exchanges, the European countries in the community 2 have formed a relatively complete value chain division of labor system after years of cooperation and trade agreements. Therefore, whether it is traditional trade or intermediate goods trade, more occurs within Europe. In the case of PTA networks, the number of communities increased over the years, rising from 4 in 2000 to 12 in 2014, and the community represented by Germany, France and other EU economies is the largest community in the networks, which is similar to the international manufacturing trade in value-added networks. Compared with the manufacturing trade network, the division of PTA communities is more dependent on geographical proximity. Most regional trade agreements on a global scale still follow the principle of geographic proximity.

**Fig 8:**
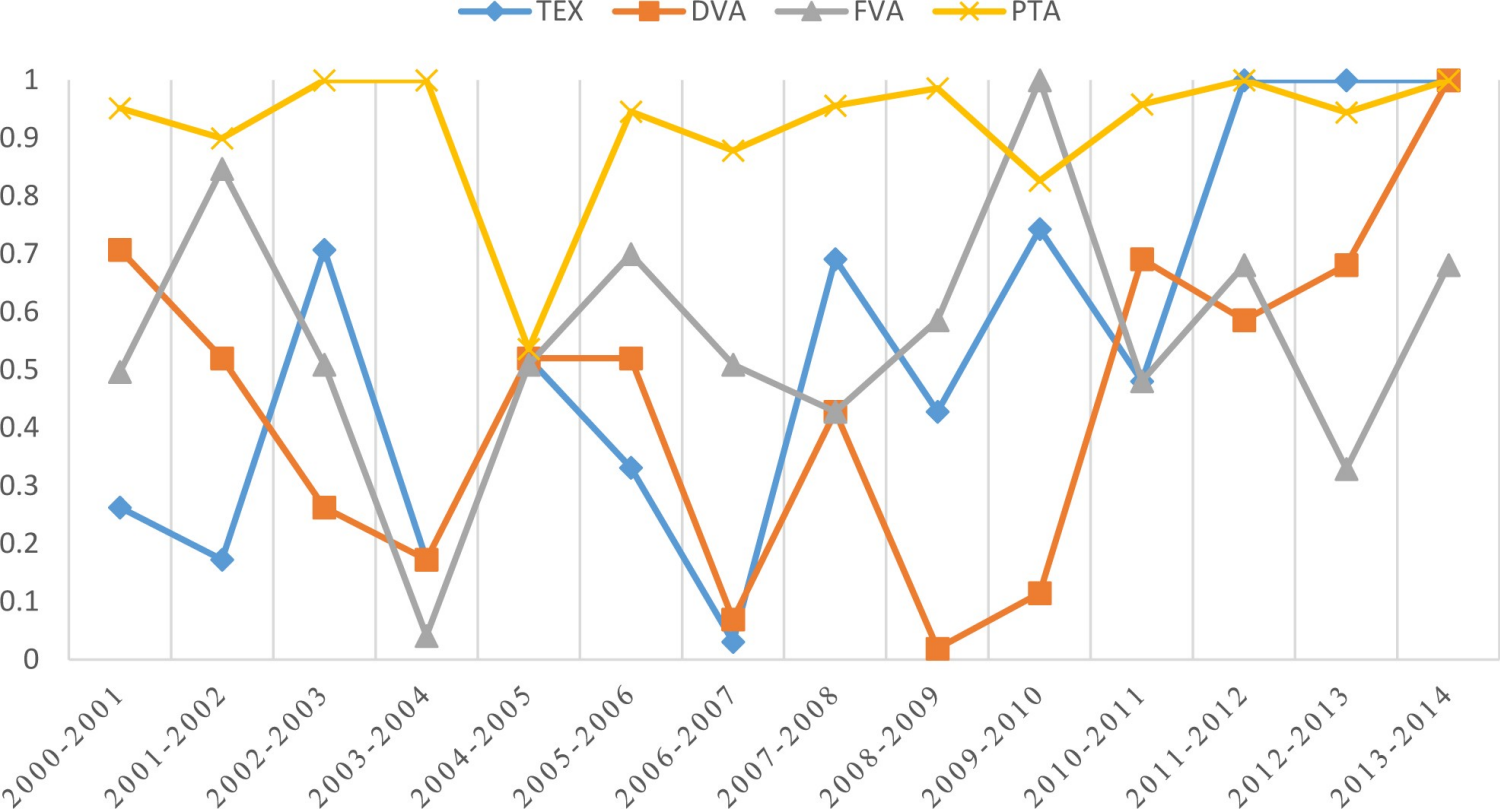
The stability of community divisions of networks over the years.

Further, to analyze the stability of the communities, as shown in Fig 8, because the relations of signing trade agreements were continuing, the community stability of the PTA networks was always at high levels from 2000 to 2014. The communities of PTA networks were relatively stable, except in 2004, when 10 economies, namely, Malta, Cyprus, Poland, Hungary, the Czech Republic, Slovakia, Slovenia, Estonia, Latvia and Lithuania, officially became members of the European Union, expanding the number of EU members from 15 to 25. The reciprocal agreement relations between EU economies made a certain difference between the community division of the PTA networks in 2004 and 2005, with the NMI value reduced to 0.54. In terms of the international manufacturing trade in value-added networks, the stability of the TEX networks increased from 0.26 in 2001 to 1 in 2014, so the international manufacturing trade in value-added networks showed a “pre-fluctuation, post-stability” trend. Specifically, the stability of the DVA network was 0.71 in 2001, and with the development of economic globalization and the participation of economies around the world in global industry specialization, the stability of the network decreased to 0.17 in 2004 and fluctuated afterwards. Affected by the financial crisis in 2009, the stability of the network reached the lowest point of 0.11, and in the post-financial crisis period, the stability of the network gradually increased and reached 1 in 2014. This means that after the outbreak of the financial crisis in 2008, the network structure of world manufacturing trade has undergone a new round of reshaping. The rise of manufacturing industries in emerging economies represented by China and India has had a huge impact on the traditional global division of labor system dominated by developed countries such as Europe and the United States. In the case of FVA networks, the average NMI over the years was 0.56, showing an overall trend of fluctuation. In 2004, the stability of the network was at the lowest of 0.04, while in 2010, it was at the highest of 1. Obviously, influenced by the development of industrial modularization and the tremendous development of transportation and communication technology, the aftermath of the fourth wave of international industrial transfer that began at the end of the last century has continued to the present. Therefore, as the added value incidental to intermediate goods from abroad, the division of FVA’s community in the period discussed in this article generally shows volatility characteristics.

**Fig 9:**
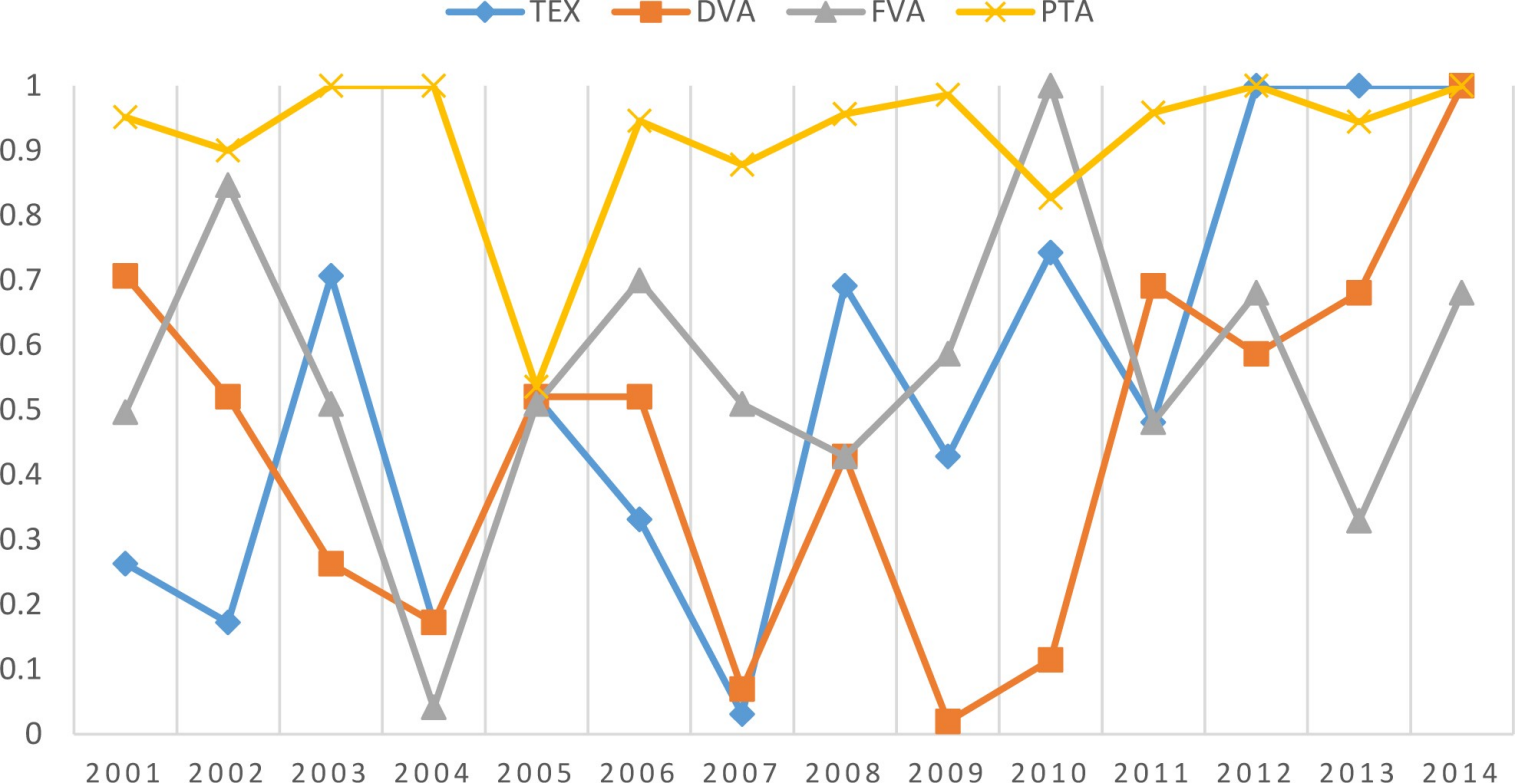
The similarity of community divisions of international manufacturing trade in value-added networks and PTA networks over the years.

Fig 9 shows the similarity of TEX, DVA and FVA networks of international manufacturing trade and PTA networks. As shown in the figure, PTA networks had a certain similarity with TEX, DVA and FVA networks of international manufacturing trade, and their corresponding average similarity over the years was 0.13, 0.12 and 0.13, respectively. Moreover, the community similarity between PTA networks and DVA networks was relatively low in the early stage but showed an overall growing trend afterwards. After 2012, the value was stable at 0.18. This means that the relationship between preferential trade agreements and the domestic value added of the manufacturing industry has increased. At the same time, the flow of DVA is consistent with the flow of final trade products. Therefore, with the diversification of mutually beneficial trade relations among countries, the development of world manufacturing trade will be more multipolar. The community similarity of the FVA network and PTA network was 0.20 in the early stage, after declining twice due to the acceleration of economic globalization after China’s accession to the WTO in 2002 and the expansion of the EU in 2004; it was stable at approximately 0.12 from 2005 to 2014. As mentioned in the division of associations above, most of the regional trade agreements around the world still follow the principle of geographic proximity. Therefore, the similarity of the FVA network and the PTA network is relatively high in the early stage, and the development process tends to stabilize after the subsequent decline. It precisely reflects the development momentum of manufacturing industries in various countries in the world, especially large multinational corporations, which are laying out industrial chains on a global scale and gradually breaking through the restrictions of geographical space.

### The analysis of QAP regression

Kimura and Lee [39] pointed out that factors such as economic scale, economic distance, common language and population scale have significant influences on the development of services trade. From the above analysis on the importance of nodes, it can also be found that the economic scale and population size have an important impact on manufacturing trade in value-added networks. Sharing a common language can affect the ease of communication in the process of trade negotiation, which will naturally affect the establishment of trade relations. In addition, through comparative analysis of the networks over the years, it can be found that economies with high node degree and node strength in PTA networks are also high-ranking in TEX and DVA networks (see appendix 2–4 for specific ranking), which suggests that preferential trade agreements are likely to affect the formation of manufacturing trade relations between economies. Therefore, using the research of Kimura and Lee [39], Duenas and Fagiolo [40] and Christen [41] for reference, we used the gravity model and selected the sum of economic scale (*gdps*) and the difference of economic scale (*gdpd*) as the economic dimension, the sum of population size (*pops*) and the difference of population size (*popd*) as the population dimension. The sum of economic and population variables means the sum of scale of two economies, while the difference of economic and population variables means the absolute value of the difference of two economies. Hence, all the all scale variables are symmetric. We also chose geographic distance (*distw*) as the physical distance dimension, common language (*lan*) as the culture dimension, preferential trade agreements (*pta*) and foreign direct investment (*fdi*) as explanatory variables to analyze their effect on manufacturing trade and value-added networks. As the flows of foreign direct investment are directed and weighted, the data of *fdi* are already relational data and asymmetric, so it’s not necessary to calculate the sum or the difference of *fdi*. In order to reduce the extreme value and heteroscedasticity of the model variables, and to facilitate the interpretation of the coefficients of the variables, this paper takes logarithm processing for all variables. The model is:

lnManu=f(lngdps,lngdpd,lnpops,lnpopd,lndistw,lnlan,lnpta,lnfdi) (8)

The economic size and population size are measured by the gross domestic product and population of each economy, and the data are from the World Development Indicators (WDI) database. Data on geographic distance and the common language are obtained from the French Research and Expertise on the World Economy database (CEPII). Foreign direct investment is measured by bilateral FDI flows over the years, and the data comes from the OECD database and the United Nations UNCTND database.

The purpose of QAP regression analysis is to explore the regression relation between multiple explanatory variable matrices and one explained variable matrix and estimate the regression results depending on the coefficients and fitted value R^2^ [42]. Both the manufacturing value-added network and PTA network constructed in this article use relational data. Since the relational data itself is data about "connections", the observations are not independent of each other and directly violate the principle of avoiding collinearity, so conventional statistical techniques such as OLS cannot be simply applied to the statistical analysis of relational data. Otherwise, the variance and standard deviation of the parameter estimates will increase, and the significance test of the variables will lose meaning, which will invalidate the prediction function of the model. The QAP method can better deal with the "multicollinearity" problem of these relational data. QAP is a non-parametric estimation method used to analyze the correlation between variables. It does not need to assume that the independent variables are independent of each other, so the results obtained are more robust than the parametric method. Table 3 shows the regression results after 1000 random permutations in 2000 and 2014.

**Table 3:** The analysis of QAP regression results.

Variable	TEX		DVA		FVA	
	2000	2014	2000	2014	2000	2014
ln(*popd*)	-1.892***	-1.183***	-0.739***	-0.758***	-0.649***	-0.725***
	(-7.2)	(-6.084)	(-9.484)	(-10.78)	(-9.285)	(-9.826)
ln(*pops*)	4.337***	2.511***	1.694***	1.694***	1.377***	1.596***
	(13.302)	(10.55)	(17.515)	(19.681)	(15.874)	(17.672)
ln(*gdpd)*	-1.563***	-1.042***	-0.436***	-0.444***	-0.276***	-0.381***
	(-8.379)	(-7.124)	(-7.881)	(-8.403)	(-5.563)	(-6.862)
ln(*gdps*)	2.701***	2.214***	0.755***	0.874***	0.538***	0.771***
	(13.441)	(14.258)	(12.67)	(15.569)	(10.067)	(13.088)
ln(*distw*)	-3.359***	-2.623***	-1.127***	-1.398***	-0.984***	-1.395***
	(-17.999)	(-19.312)	(-20.371)	(-28.464)	(-19.81)	(-27.066)
ln(*lan*)	2.299*	-0.091	1.394***	0.835**	1.34***	1.004***
	(3.316)	(-0.183)	(6.781)	(4.622)	(7.265)	(5.302)
ln(*pta*)	0.383*	-0.055	0.216***	-0.051*	0.221***	-0.043
	(3.731)	(-1.164)	(7.11)	(-2.999)	(8.115)	(-2.429)
ln(fdi)	0.357	0.274	0.629***	0.299**	0.693***	0.322**
	(0.871)	(0.839)	(5.168)	(2.53)	(6.345)	(2.599)
R^2^	0.4286	0.5171	0.5009	0.5841	0.448	0.504
Adjusted R^2^	0.4261	0.5149	0.4986	0.5822	0.4455	0.5018
N	1806	1806	1806	1806	1806	1806

The data in Table 3 show that the goodness of fit of TEX, DVA and FVA networks of manufacturing trade in 2014 are 0.5149, 0.5822 and 0.5018, respectively, which means that the variables included in the model can explain 51.49%, 58.22% and 50.18% of the corresponding network structure changes. The sample volumes are all 1806, indicating that the model includes a 43×43 matrix composed of 43 economies included in the WIOD input-output data (the diagonal elements of the matrix are 0; that is, there is no self-loop in the networks).

As shown in Table 3, the impacts of the economic scale, population size, geographical distance, common language and foreign direct investment on international manufacturing trade networks are in line with expectations. From the perspective of economics, the sum of the economic scale of two economies has a positive effect on international manufacturing trade in value-added networks, while the effect of the difference in economic scale is negative, and the regression coefficient is significant at the 1% level, which indicates that the larger the overall economic scale of the trading partners is, the stronger the manufacturing trade links between them will be. The larger economic scale gap hinders the development of manufacturing trade, and the smaller the economic scale difference between economies is, the stronger the trade links will be.

In terms of population, the sum of population size can promote the international manufacturing trade networks, but the difference of population size has a negative effect on international manufacturing trade networks, which means that the larger the total population size of two economies is, the more favorable it will be for the manufacturing trade in value-added, and the larger the gap in population size is, the worse the development of manufacturing trade between the two economies will be.

Geographical distance inhibits international manufacturing trade networks, but common language plays a role in promoting international manufacturing trade networks, which indicates that the trade relations between two economies will be stronger if they are close geographically or share a common language. The impact of foreign direct investment on the total manufacturing trade is not significant, but it has a significant positive correlation with the domestic value added (DVA) of final exports and the foreign value added (FVA) of domestic exports in the manufacturing trade. This shows that the frequent exchanges of bilateral direct investment make the manufacturing intermediate product trade market between partner countries more active, but it has little impact on the final traded product. In developing countries, trade in intermediate goods has developed rapidly, and east Asian countries, in particular, are much more dependent on this new international division of production than are North American and European countries. Cerina et al. [43] and João, et al. [44] identify the rising importance of China in GVCs. Ferrarini [45] uses international trade data on products classified as parts and components to quantify vertical trade among countries and applies visualization tools to map the resulting global network of vertical trade, highlighting the rise of China and the importance of the automotive and electronics sectors in GVCs. In fact, the trade in intermediate goods in China has grown rapidly in the past two decades, especially in the industries of electronic components, mechanical equipment and communication. This was also the period of China’s accession to the World Trade Organization and the rapid development of the country’s reform and opening-up. China is one of the world’s largest recipients of foreign investment. At the same time, China’s trade in intermediate goods has grown rapidly with the increase in China’s utilization of foreign capital. China’s import and export in intermediate products has become an important part of China’s international trade.

For the core variable in this paper, the effect of the PTA network on the international manufacturing trade networks changed from positive in 2000 to negative in 2014. Furthermore, QAP regression was carried out in each year from 2000 to 2014 according to formula (7) in this paper, and the corresponding regression coefficients of PTA were extracted, as shown in Table 4.

**Table 4:** The regression coefficients of PTA in 2000–2014.

Year	Variable	TEX	DVA	FVA
2000	ln(*pta*)	0.383*(3.731)	0.216***(7.11)	0.221***(8.115)
2001	ln(*pta*)	0.318(3.143)	0.19***(6.47)	0.209***(7.77)
2002	ln(*pta*)	0.221(2.274)	0.154**(5.326)	0.169***(6.337)
2003	ln(*pta*)	0.239(2.588)	0.153**(5.463)	0.162***(6.093)
2004	ln(*pta*)	-0.166*(-2.719)	-0.038(-1.982)	-0.016(-0.832)
2005	ln(*pta*)	-0.172*(-2.902)	-0.04(-2.056)	-0.001(-0.047)
2006	ln(pta)	-0.172*(-3.028)	-0.04(-2.133)	-0.001(-0.035)
2007	ln(*pta*)	-0.176**(-3.451)	-0.066**(-3.688)	-0.03(-1.651)
2008	ln(*pta*)	-0.138*(-2.766)	-0.059*(-3.312)	-0.039(-2.161)
2009	ln(*pta*)	-0.169**(-3.134)	-0.058**(-3.217)	-0.035(-1.884)
2010	ln(*pta*)	-0.05(-0.978)	-0.048*(-2.685)	-0.03(-1.658)
2011	ln(*pta*)	-0.112(-2.338)	-0.052*(-2.982)	-0.034(-1.875)
2012	ln(*pta*)	-0.082(-1.72)	-0.052*(-2.959)	-0.036(-1.965)
2013	ln(pta)	-0.126**(-2.704)	-0.062**(-3.701)	-0.054(-3.072)
2014	ln(*pta*)	-0.055(-1.164)	-0.051*(-2.999)	-0.043(-2.429)

From the QAP regression coefficients of the PTAs from 2000–2014 in the table, we can see that the effect of PTA networks on manufacturing trade networks varied greatly over the years, and there was a significant turning point in 2004. Before 2004, the PTA networks had a promoting effect on the TEX, DVA and FVA networks, and the absolute values of the coefficients declined year by year. However, in 2004 and the years after that, the effect of PTA networks on TEX, DVA and FVA networks became negative, and the absolute values increased continuously from 2004 to 2008. The impact of PTA on international manufacturing trade networks can be divided into two stages. Before 2004, there was a significant positive effect of PTA networks on DVA and FVA networks, indicating that regional preferential trade agreements can significantly promote manufacturing trade among participating economies, especially the trade in value-added in the form of DVA and FVA. However, after 2004, the effect of PTA networks on the DVA and FVA networks became negative, which means that after 2004, regional preferential trade agreements may inhibit the manufacturing trade between economies. In particular, from 2004 to 2008, the absolute values of the negative coefficients increased year by year, indicating that the inhibiting effect of PTAs became greater.

Considering that there might be a time lag between the signing and effect of PTAs, we replaced the variable of PTAs with the variable of the lag of PTAs (5 years) as a robust check to explore whether the previously signed PTAs have similar effect on manufacturing trade, and the results are listed in Table 5. It can be seen that the promoting effect of PTAs are mainly in 2000 to 2003, and therefore the turning point do appeared in 2004.

**Table 5:** The regression coefficients of the lag of the PTAs (5 years) in 2000–2009.

Year	Variable	TEX	DVA	FVA
2000	ln(lag*pta*)	0.20681(2.426)	0.14157**(5.149)	0.15165***(5.618)
2001	ln(lag*pta*)	0.15954(1.956)	0.12495**(4.63)	0.12947**(4.792)
2002	ln(lag*pta*)	0.06332(0.84)	0.09693*(3.676)	0.11086*(4.157)
2003	ln(lag*pta*)	0.10657(1.461)	0.10221*(3.925)	0.10486*(3.959)
2004	ln(lag*pta*)	-0.1455*(-2.575)	-0.03876(-2.04)	-0.00732(-0.377)
2005	ln(lag*pta*)	-0.07932(-1.478)	-0.03748(-2.015)	-0.00268(-0.14)
2006	ln(lag*pta*)	-0.1176(-2.324)	-0.04831(-2.634)	-0.01313(-0.681)
2007	ln(lag*pta*)	-0.12305*(-2.55)	-0.0659**(-3.68)	-0.04789(-2.574)
2008	ln(lag*pta*)	-0.14734**(-3)	-0.06846**(-3.858)	-0.05288(-2.864)
2009	ln(lag*pta*)	-0.06867(-1.391)	-0.055*(-3.08)	-0.03987(-2.127)

The above findings further confirm the views of Cheng & Tsai [46] and Martinez-zarzoso et al. [47] that the impact of regional trade agreements on trade flows between participating economies is dynamic, phased and not static. Also, the effect of PTAs on trade in value-added also depends on the depth of trade agreements and it may be heterogeneous in different industries or different economies [48], and some provisions such as competition policy and investment can influence the relation between PTA and trade in value-added as well [49].

Combining the progressive internationalized theory and relevant international background, we believe that the above changes in PTA network coefficients can be attributed to the following three reasons. First, at the overall network level, due to the expansion of EU members from 15 to 25 in 2004, the number of nodes in networks rose from 21 to 32, and the number of edges nearly doubled from 188 to 443. At this stage, economic globalization accelerated, and economies around the world started the trend of trade liberalization. When choosing trading partners, economies no longer limited trade to a few countries that had signed trade agreements with them, they looked for the most suitable trading partners around the world to maximize their comparative advantages. Therefore, the trade creation effect brought by regional trade agreements was absorbed by the trend of trade liberalization around the world, and the promotive effect of trade agreements became increasingly nonsignificant, resulting in a turning point in the correlation between PTAs and manufacturing trade in value-added around 2004. In recent years, the United States has dropped out of the Trans-Pacific Partnership Agreement (TPP), the Paris Agreement on climate change, the United Nations Educational, Scientific and Cultural Organization and the Joint Comprehensive Plan of Action, and the United Kingdom exited from the European Union. These actions are strong evidence of the weakening and even reverse effects of PTA trade creation.

Second, in the field of trade in goods, tariff and non-tariff measures are important indicators to measure the degree of a country’s trade openness. The reduction of tariff and non-tariff measures is bound to have an important impact on manufacturing trade because manufacturing trade is the main form of trade in goods. As the world’s factory, China’s large-scale gradual reduction of tariff and non-tariff measures after its accession to the WTO in 2001 had an important impact on global manufacturing trade. After China’s accession to the WTO, the average tariff level of China gradually reduced from 15.3% to 10.4% in 2004. In terms of non-tariff measures, before China joined the WTO, it implemented an import quota and license administration for 377 products (according to the 8-digit tax code). By 2004, the import quota and license administration for 342 products had been cancelled, transforming to tariff quota or automatic import license, which conforms to WTO rules. The effect of tariff and non-tariff measures on manufacturing trade is obviously similar to the effect of PTA, and the promotive effect of the reduction of tariff and non-tariff measures is even more direct and effective. Therefore, around 2004, the gradual implementation of China’s commitments to increase trade openness after its accession to the WTO also absorbed part of the positive effect of PTAs on manufacturing trade, making its regression coefficients decrease continuously.

Third, from the perspective of multinational companies, according to progressive internationalization theory, most multinational companies choose progressive internationalization strategies when trying to enter foreign markets. In particular, the manufacturing industry tends to start with export, become familiar with the market of the host economy through export, accumulate a certain consumer base, and then change from export to direct investment in the host economy. Obviously, after signing bilateral or multilateral PTAs, most companies will be attracted to preferential policies and then increase exports to host economies that have signed preferential trade agreements. After a period of accumulation and exploration, to achieve higher efficiency and lower cost, companies are likely to invest directly in the host economy and replace exports with local production. As a result, the trade creation effect of preferential trade agreements is gradually transformed into the effect of foreign investment, and in this way, the positive impact of PTAs on the manufacturing trade networks is continuously reduced and even turns negative.

## Conclusions

Based on the trade in value-added accounting method, this paper has divided international manufacturing gross trade flows into domestic value-added (DVA), foreign value-added (FVA), returning domestic value-added (RDV) and purely double-counted trade in intermediate goods (PDC) and adopted network modeling to measure the international manufacturing trade in value-added networks. Moreover, we have used the data of preferential trade agreements data of the World Bank to build PTA networks and explored the relations between international manufacturing trade in value-added networks and PTA networks from the perspective of overall features, community structure and influencing factors. The following major conclusions have been obtained:

1. There was an obvious agglomeration effect in the international manufacturing trade in value-added networks and PTA networks over the years, and the size of networks grew rapidly. With the deepening of international specialization, economies around the world became increasingly embedded in the global value chain, and the reciprocal trade relations among economies increased as well. In addition, the financial crisis in 2008 and the expansion of the EU in 2004 had a significant impact on the international manufacturing trade in value-added trade networks and PTA networks.

2. Over the years, all the TEX, DVA and FVA networks of international manufacturing trade in value-added can be divided into two communities: a community represented by EU economies, such as Germany, France and other European economies, and a community represented by emerging economies, such as China and India, as well as North America and East Asia. Moreover, the number of communities in PTA networks increased overall. However, due to the continuity of trade agreement relations in the PTA network, the community division of PTA networks was quite stable. The PTA network and TEX, DVA and FVA networks of international manufacturing trade have a certain similarity, and the community division of PTA networks and DVA networks show a growing trend.

3. The economic scale, the difference in economic scale, population size, the difference in population size, geographical distance, foreign direct investment and common language are the main factors affecting the manufacturing trade networks and manufacturing trade in value-added networks. The economic scale, population size and common language have a significant promotive effect on the manufacturing trade networks and manufacturing trade in value-added networks, while the difference in economic scale, the difference in population size and geographic distance inhibit the development of manufacturing trade and manufacturing trade in value-added. Specifically, by comparing the regression coefficients of PTA networks, we found that, affected by economic globalization, the expansion of the EU and enterprise internationalization, the year 2004 appeared to represent a significant turning point in the relation between PTA networks and trade in value-added networks. Before 2004, the correlation of PTA networks with DVA and FVA networks was significantly positive, which means that from 2000 to 2004, PTA networks had a promotive effect on trade in value-added in the form of DVA and FVA, but the effect gradually decreased. After 2004, the effects of PTA networks on TEX, DVA and FVA networks were negative.

According to the above conclusions, in order to positively promote the development of global value chain, and help to solve the game and trade frictions among countries such as Sino-US under the new historical background of reshaping international trade order, the following suggestions are proposed in this paper. First, the shaping of trade in value-added networks, especially the early stage of trade in value-added, mainly depends on the arrangement of PTA. Therefore, economies should make active use of the reciprocal relations in the networks to strengthen trade ties with each other, and vigorously promote multilateral trade liberalization negotiations. Second, the results of community division indicate that the statuses and roles of different economies are heterogeneous, hence when economies seek the upgrading path of their manufacturing industries, they should take their roles in the trade in value-added networks and PTA networks into account, and select the trade partners and competitive industries wisely. Third, the manufacturing development strategy should be organically combined with PTA development strategy, and manufacturing trade should be cultivated in a selective and directional way according to heterogeneous features of economic, humanistic and geographical relations with other economies.

## Supporting information

S1 Table The internationalized domain names of economies in this paper. (PDF)

S1 Fig The subdivisions of DVA in 2014. (TIF)

S2 Fig The subdivisions of FVA in 2014. (TIF)

S1 Data Subdivision data of bilateral manufacturing trade in 2000. (XLSX)

S2 Data Subdivision data of bilateral manufacturing trade in 2014. (XLSX)
