# Supplementary material for: The influence of regional preferential trade agreements on international manufacturing trade in value-added: Based on the complex network method
Source: PLoS One. 2021 Feb 19;16(2):e0246250. doi: 10.1371/journal.pone.0246250 (PMC7894938; doi:10.1371/journal.pone.0246250)
Supplement: S1 Table — (PDF) [file pone.0246250.s001.pdf]

S1 Table. The internationalized domain names of economies in this paper

Economy	IDN	Economy	IDN	Economy	IDN
Australia	AUS	France	FRA	Malta	MLT
Austria	AUT	The United Kingdom	GBR	Netherlands	NLD
Belgium	BEL	Greece	GRC	Norway	NOR
Bulgaria	BGR	Croatia	HRV	Poland	POL
Brazil	BRA	Hungary	HUN	Portugal	PRT
Canada	CAN	India	IDN	Romania	ROU
Switzerland	CHE	Indonesia	IND	Russia	RUS
China	CHN	Ireland	IRL	Slovakia	SVK
Cyprus	CYP	Italy	ITA	Slovenia	SVN
Czech Republic	CZE	Japan	JPN	Sweden	SWE
Germany	DEU	Korea	KOR	Turkey	TUR
Denmark	DNK	Lithuania	LTU	Taiwan, China	TWN
Spain	ESP	Luxembourg	LUX	the United State	USA
Estonia	EST	Latvia	LVA		
Finland	FIN	Mexico	MEX		
