# Supplementary figures and images for: The influence of regional preferential trade agreements on international manufacturing trade in value-added: Based on the complex network method
Source: PLoS One. 2021 Feb 19;16(2):e0246250. doi: 10.1371/journal.pone.0246250 (PMC7894938; doi:10.1371/journal.pone.0246250)

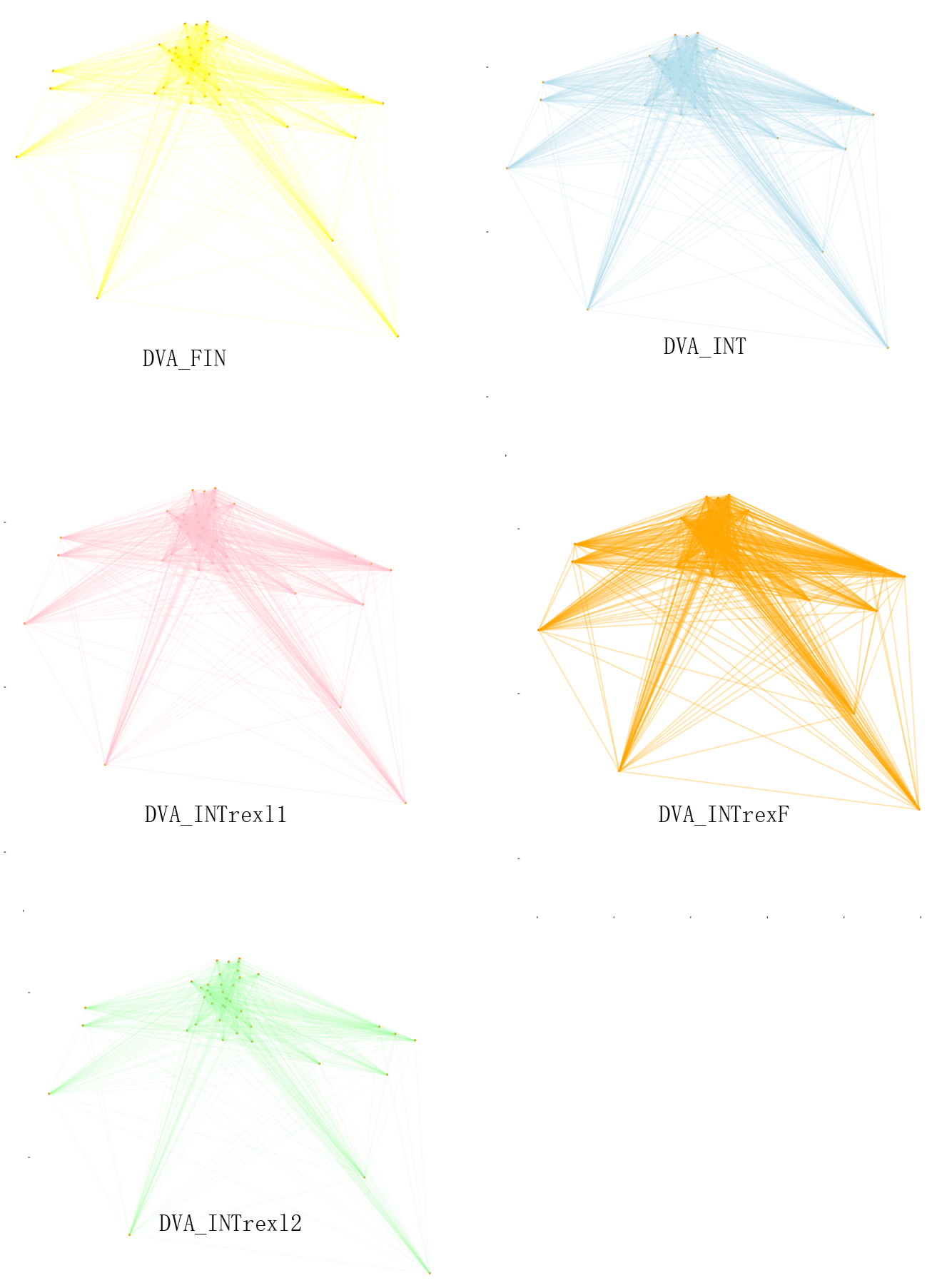

Supplement: S1 Fig — (TIF) [file pone.0246250.s002.tif]

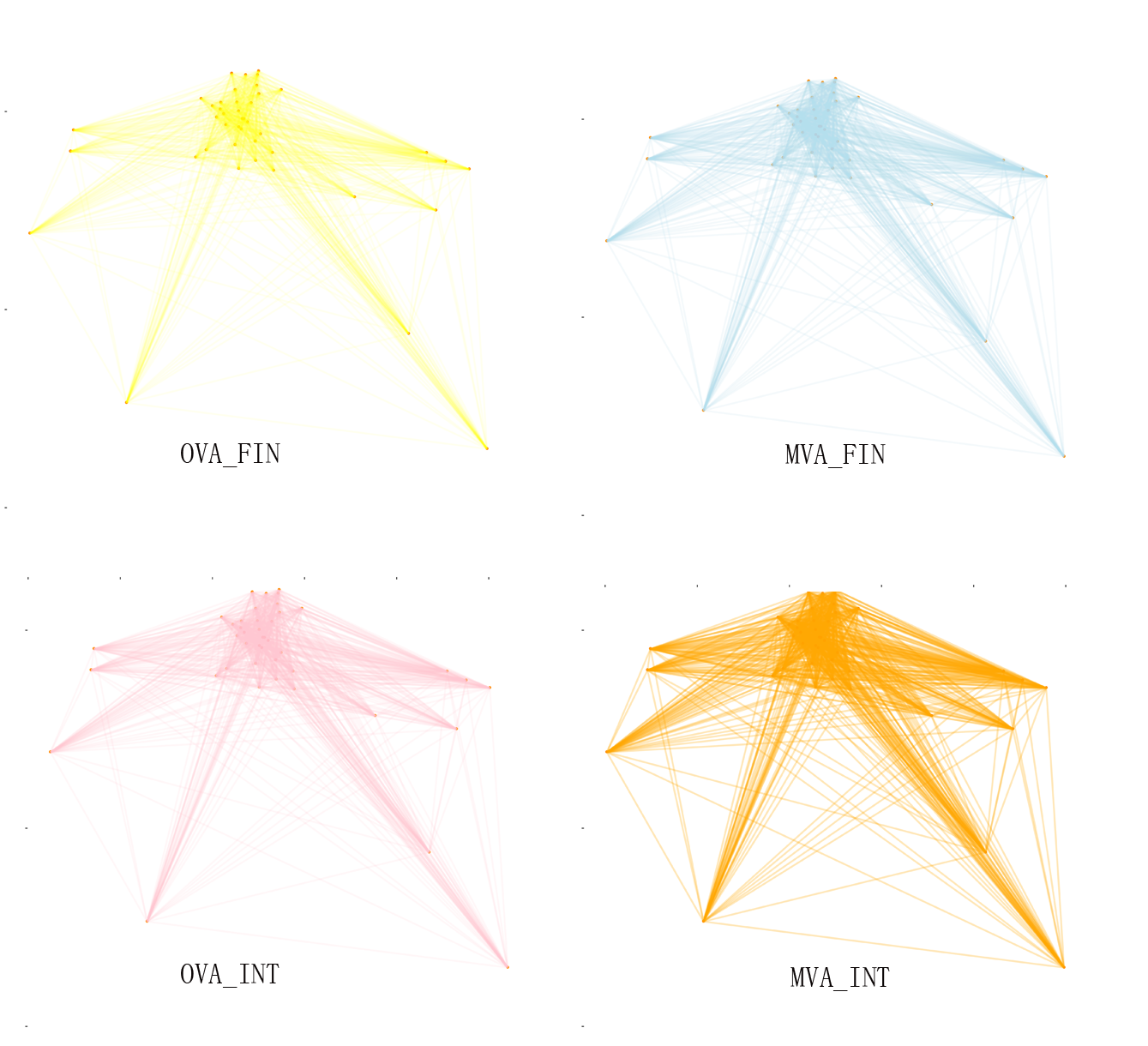

Supplement: S2 Fig — (TIF) [file pone.0246250.s003.tif]
